# Brecha de género y etnia en enfermedad carotídea: experiencia de un hospital nacional peruano

**DOI:** 10.47487/apcyccv.v6i3.526

**Published:** 2025-09-24

**Authors:** Georgette Vetanzo-Sánchez, Alexander Brioso-Cevallos

**Affiliations:** 1 Unidad de Cirugía Vascular y Endovascular. Servicio de Cirugía Cardiovascular. Hospital Nacional Guillermo Almenara Irigoyen. Lima, Perú. Unidad de Cirugía Vascular y Endovascular Servicio de Cirugía Cardiovascular Hospital Nacional Guillermo Almenara Irigoyen Lima Perú

*Sr. Editor:*

La enfermedad carotídea aterosclerótica es una causa significativa de eventos cerebrovasculares a nivel mundial ^1^. Las recomendaciones sobre el manejo de esta patología, según las guías internacionales, se presentan de forma global, sin tener en cuenta las diferencias de ciertos grupos. Sin embargo, aunque se mencionan las diferencias entre el sexo y la raza en la prevalencia de la enfermedad, aún existe una notable escasez de estudios centrados en su prevalencia, progresión y manejo en mujeres latinas ^1^^,^^2^^,^^3^.

Diversas investigaciones han mostrado las diferencias étnicas en la prevalencia y características de la enfermedad carotídea ^4^. Un ejemplo de ello es el estudio HELIUS ^5^, en los Países Bajos, donde se encontraron variaciones significativas en el grosor de la íntima-media carotídea y la menor presencia de placas entre distintos grupos étnicos, resaltando la necesidad de considerar la etnia en la evaluación del riesgo cardiovascular. Respecto a la población latinoamericana, se puede mencionar un estudio realizado en una población mestiza mexicana donde se identificó una asociación entre el polimorfismo del gen PCSK9 y el aumento del grosor de la íntima-media carotídea en individuos asintomáticos; esto resalta la importancia de los factores genéticos específicos de esta población ^6^.

Del mismo modo, investigaciones en mujeres hispanas en Estados Unidos han mostrado una menor prevalencia de placas carotídeas en comparación con mujeres blancas, a pesar de una mayor carga de factores de riesgo cardiovascular. Este fenómeno, conocido como la «paradoja hispana», sugiere la existencia de mecanismos protectores aún no completamente explicados ^2^.

Las guías actuales de manejo de la enfermedad carotídea se basan predominantemente en estudios realizados en poblaciones blancas, sin reflejar adecuadamente las necesidades y características de las mujeres latinas. No solo debemos considerar los factores propios del sexo y la etnia de una mujer latina, sino que también debemos tener en cuenta otros aspectos, como las inequidades en el acceso a la salud. Por ejemplo, un estudio en Estados Unidos demostró que los pacientes negros y las mujeres tenían tasas mucho menores de endarterectomías carotídeas tras un evento cerebrovascular o accidente isquémico transitorio ^1^^,^^4^^,^^7^.

Nuestro hospital es un centro de referencia a nivel nacional en Lima, Perú, y entre 2023 y 2024 se realizaron 20 cirugías de endarterectomía carotídea, siendo 4 mujeres y 16 hombres; esto coincide con la literatura universal, donde esta patología es más frecuente en el grupo masculino. El promedio de edad fue de 71,7 años en mujeres y 74,9 años en varones. Asimismo, la comorbilidad más frecuente fue la hipertensión arterial en ambos géneros, lo cual coincide con otros estudios, y el antecedente de un evento cerebrovascular fue más frecuente en los varones (Figura 1) ^(^^1^. Aunque el número de cirugías realizadas anualmente se compara con otros centros a nivel latinoamericano, como en Brasil, donde mencionan que operan en promedio a siete pacientes por año, en nuestro centro se encontró que solo el 20% eran mujeres, a diferencia de Brasil, donde reportaron aproximadamente el 30% ^8^. Esto recalca la importancia de analizar de manera particular a la población femenina latina.

**Figura 1:**
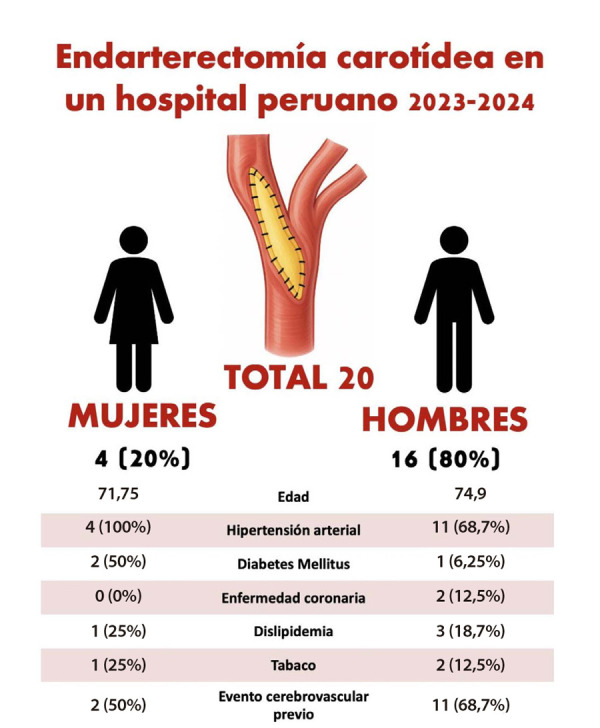
Características de los pacientes operados de endarterectomía carotidea 2023-2024, Hospital Nacional Guillermo Almenara Irigoyen. Lima, Perú.

Es necesario fomentar investigaciones que aborden esta brecha de conocimiento, considerando las particularidades genéticas, culturales y socioeconómicas de las mujeres latinas. Con ello, lograríamos una representación equitativa en las investigaciones y así desarrollar estrategias de prevención y tratamiento más efectivas y personalizadas para esta población.

## Financiamiento:

Autofinanciado
